# A fungus infected environment does not alter the behaviour of foraging ants

**DOI:** 10.1038/s41598-021-02817-8

**Published:** 2021-12-07

**Authors:** Hugo Pereira, Romain Willeput, Claire Detrain

**Affiliations:** Unit of Social Ecology, Université Libre de Bruxelles, CP 231, 50 avenue F. Roosevelt, 1050 Brussels, Belgium

**Keywords:** Behavioural ecology, Pathogens, Animal behaviour

## Abstract

Eusocial insects are exposed to a wide range of pathogens while foraging outside their nest. We know that opportunistic scavenging ants are able to assess the sanitary state of food and to discriminate a prey which died from infection by the entomopathogenic fungus *Metarhizium brunneum.* Here, we investigate whether a contamination of the environment can also influence the behaviour of foragers, both at the individual and collective level. In a Y-maze, *Myrmica rubra* ants had the choice to forage on two prey patches, one of which containing sporulating items. Unexpectedly, the nearby presence of sporulating bodies did not deter foragers nor prevent them from retrieving palatable prey. Ant colonies exploited both prey patches equally, without further mortality resulting from foraging on the contaminated area. Thus, a contamination of the environment did not prompt an active avoidance by foragers of which the activity depended primarily on the food characteristics. Generalist entomopathogenic fungi such as *M. brunneum* in the area around the nest appear more to be of a nuisance to ant foragers than a major selective force driving them to adopt avoidance strategies. We discuss the cost–benefit balance derived from the fine-tuning of strategies of pathogen avoidance in ants.

## Introduction

Insect societies prove to be highly efficient to discover and exploit available food resources in their environment^1^. Food exploitation relies on the cooperation between the foragers, which explore nest surroundings and retrieve the discovered resources, and the internal workers, which store, process and distribute food inside the nest. In this cooperative exploitation of environmental resources, foragers play a key role because they regulate the dynamics of nestmate recruitment and orient the choice made by the whole colony among several alternative resources. The level of exploitation of a newly discovered food source will then depend on the decision of successful foragers to actively recruit nestmates by direct contacts and/or by the laying a pheromone trail on their way back to the nest^2,3^. The release of a recruitment signal by foragers, which will trigger a collective exploitation of food, depends on numerous factors such as the carbohydrate concentration of a food droplet^4–7^, the quantity of food^8–10^, its nature (e.g. prey or sugar solution)^11–13^, its distance from the nest^14,15^ but also the colony's current nutritional status^13,16–18^. A fine-tuning of recruitment signals by foragers allows an efficient adjustment of food exploitation to the current needs of the colony and usually leads the colony to choose the most rewarding sources (but see^19,20^).

Besides the social information mediated by pheromone trails, ant individuals improve their navigational skills over successive foraging trips^21^ and may eventually develop a fidelity to stable and rewarding food sites. Such a site fidelity has been observed in many different ants such as ponerine ants (*Dinoponera quadriceps*^22^), harvester ants (*Pogonomyrmex occidentalis*^15^; *Pogonomyrmex barbatus*^23)^, formicine ants (F*ormica schaufussi*^24^; *Lasius niger*^20,25^) and myrmicine ants (*Myrmica rubra*^26^). Route learning coupled to fidelity to a foraging site minimizes the travel time, reduces the likelihood of foragers to be lost, speeds up the dynamics of food exploitation and eventually favours the choice of this site by the whole colony. Spatial fidelity of foragers increases with food abundance^23,27^, and depends on food nature with a greater tendency to thoroughly explore an area where they previously found carbohydrates instead of insect prey^24^.

A large number of studies have thus demonstrated that the foraging behaviour of ants closely depends on the characteristics of available resources as well as on the current needs of the colony. Far less is known about the impact of the surrounding environment as a driving force that can shape ants’ foraging responses. As regards to the abiotic environment, the soil substrate can modify the collective choice of a foraging route^28^ and the ambient temperature can influence the running speed and the searching behaviour of foragers^29^. Besides, biotic factors such as the presence of predators or competitors are known to act upon ants’ foraging, both at the individual and collective level. For example, predators (*Formica sanguinea*) near a food source influence the choice of *Lasius pallitarsis* and *Myrmica incompleta* foragers, which abandon a rich source when the risks incurred outweigh the expected energy benefits^30^. Likewise, competitors can alter foraging in different ways by either making the ants avoiding the location^31^, by increasing recruitment to food^32^ or by eliciting no response^33^.

In addition to competitors and predators, parasites near a food source or on foraging routes represent another major risk, not only for the foragers themselves but for the colony as a whole. For example, the presence of parasitoid flies near the ant nest leads to a decrease in its foraging activity (in *Solenopsis invicta*^34^ and *Pheidole dentata*^35^). Likewise, microparasites such as fungi, virus or bacteria could shape the strategies of food exploitation in ants. These microparasites are assumed to exert a strong selective pressure on the insect societies, namely because they are composed of genetically-related individuals that live at high densities in a confined nest^36^. To counter sanitary risks, insect societies rely on social immunity which encompasses adaptive cooperative defences against pathogens having evolved at the physiological, behavioural and organisational levels^37^. Social immunity is namely achieved through grooming^38^, use of antimicrobial substances on nest materials^39^, discarding of waste^40–42^, removal of dead nestmates^43,44^ or self-exclusion of moribund workers^45,46^. A first line of defence against pathogens consists in the prevention of their entry inside the colony, notably by avoiding to retrieve contaminated food items. This sanitary control is particularly important for scavenging ant species since dying prey or insect cadavers commonly host a wide range of entomopathogenic microorganisms.

Since foragers can become vectors of entomopathogens from the outside and may contaminate other workers inside the nest, one may expect them to avoid exploring health-hazardous areas or feeding on infectious food items. A few studies on eusocial insects demonstrated that foragers actually adapt their behaviour in order to limit sanitary risks. In this respect, one must differentiate the sanitary risks of feeding on pathogen-bearing food items and those incurred by foraging in their proximity. For instance, *Bombus terrestris* foragers avoid collecting nectar on flowers contaminated with a protozoan flagellate parasite *Crithidia bombi*^47^. Likewise, several ant species do not collect food items that are covered with *Metarhizium brunneum* entomopathogenic conidia^48–50^. These few studies demonstrate that the sanitary state of a food resource can act upon its level of exploitation. Far less is known on how the contamination of the surrounding environment may impact ants’ foraging. Indeed, ants are likely to get infected when they encounter fungal conidia scattered over the soil or released by a sporulating cadaver located on the foraged area. Studying the avoidance of contaminated zones will provide ecologically-relevant insights on the ability of ants to show prophylactic behaviour and to limit sanitary risks while foraging. Furthermore, this raises questions on whether ants have evolved avoidance behaviour to delayed sanitary risks. While predation or competition directly impacts foragers through injuries or death, a contamination of the environment by pathogens has delayed effects on workers that may become ill several days after being exposed to microparasites.

In this study, we investigate whether the propensity of *Myrmica rubra* ants to retrieve food items and/or to return to a resource will depend on the sanitary risks associated to the foraged areas. *Myrmica rubra* is an opportunistic omnivorous species whose workers collect aphid honeydew but also regularly forage on living or dead insects. *M. rubra* foragers are known to avoid sporulating prey^48^ but it remains unknown whether they are also able to assess the sanitary risks associated to the site of food exploitation. At the individual level, we will investigate in a Y-maze, whether the vicinity of *Metarizium brunneum* sporulating insects may influence the retrieval rate of non-infected palatable prey as well as the spatial fidelity of workers over their successive foraging trips. At the colony level, we will compare the dynamics of ant flows (i.e. the number of foragers entering each foraging area over successive 5-min time intervals) and the potential selection of a food source depending on the nearby presence of sporulating cadavers. We assume that a time-and spatially-limited search by individual foragers will occur over conidia-contaminated areas since it would reduce the sanitary risks incurred.

## Results

### Individual response of foragers

When making their first trip to the Y maze, individuals did not preferentially explore the left or the right platform (N_left_ = 18; N_right_ = 27; binomial test: *P* = 0.23), suggesting there was no orientation bias due to external factors in the surrounding environment. Furthermore, exploring ants did not seem to be repelled nor attracted from a distance by sporulating items. Indeed, foragers that first explored the uninfected foraging area (UFA) were as numerous as those that first headed towards the contaminated foraging area (CFA) (N_UFA_ = 22 ants and N_CFA_ = 23 ants, binomial test: *P* = 1). During this first trip, we observed a very small number of grooming behaviours (N = 14 in total), which occurred equally on the UFA and on the CFA (7 grooming events on each area) and which lasted as long on the UFA (m = 9 s [5; 21]; N = 7) as on the CFA (m = 8 s [4; 11]; N = 7) (Mann-Whitney test: MW = 31.5; *P* = 0.4).

**Figure 1:**
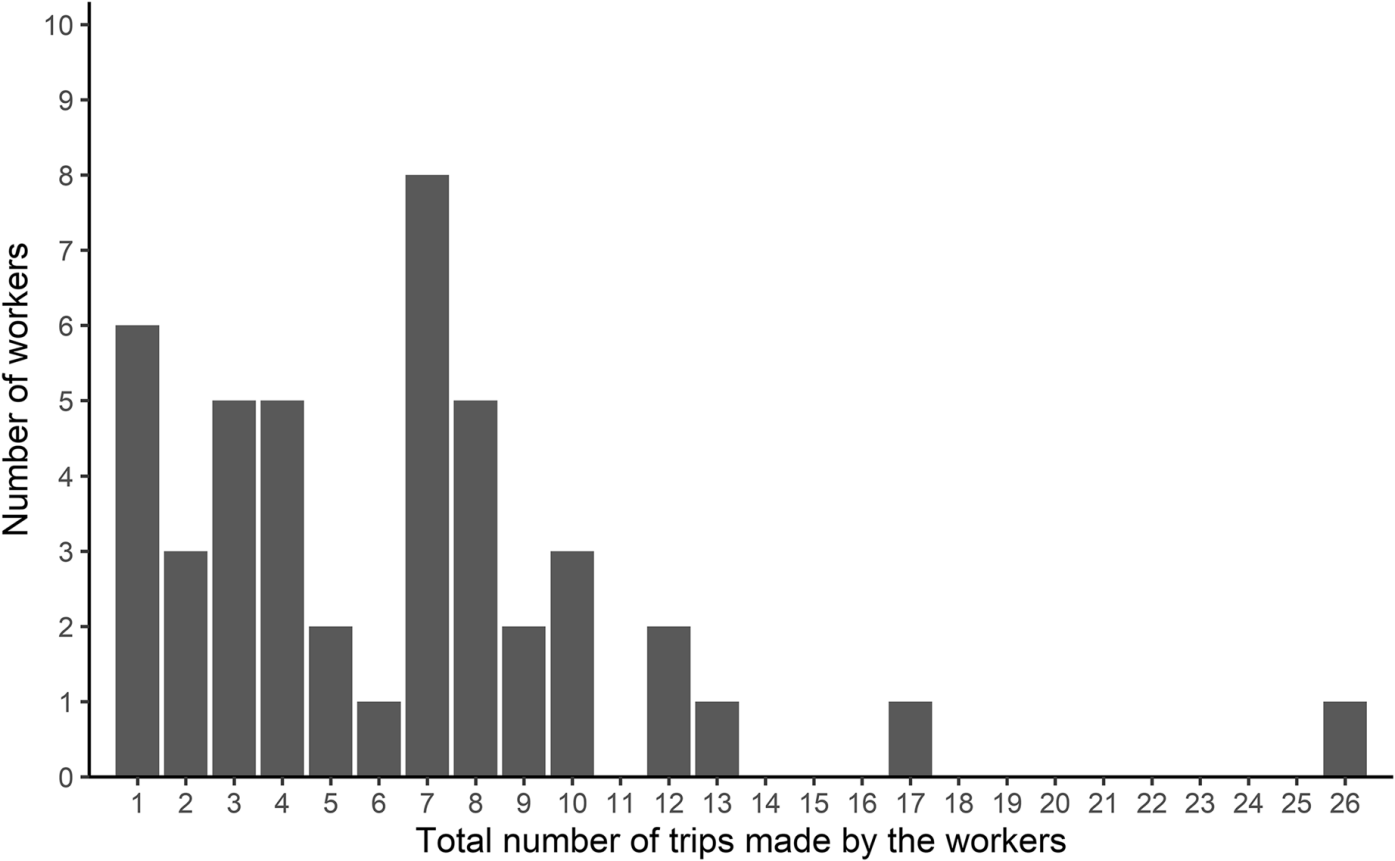
Individual experiment: distribution of the number of workers as a function of the total number of trips they made toward the Y-maze (N = 45).

During the allocated time (i.e. 90 min), individual foragers made a highly variable number of trips towards the Y-maze, which ranged from 1 to 26 trips (Fig. 1). Only six workers (out of 45 workers observed) made a single trip and did not show up for a second time over the Y-maze. Most foragers (39 of 45 workers) returned several times on the Y-maze, resulting in a total cumulated number of 287 trips observed between the nest and the foraging platforms. In most cases (259 out of 287 trips), the worker visited only one of the two foraging areas. In the few cases (28 out of 287 trips) where the ant visited the two platforms, this prolonged exploration did not result from a redirection of the ants’ activity from the contaminated area towards the uncontaminated one. Indeed, these trips were as likely to start with a first visit to the UFA (N_UFA_ = 17 trips) as to the CFA (N_CFA_ = 11 trips) (binomial test: *P* = 0.34). We also found that the propensity for an individual to return several times to the Y-maze was not altered by its exposure to a contaminated environment. Indeed, there was no significant relationship between the proportion of visits made by a forager to the CFA and its total number of foraging trips (Spearman's test: rs = 0.029; *P* = 0.85; N = 45; see Supplementary Fig. 1). Overall, foragers stayed on each foraging platform for a similar amount of time regardless of the sanitary state of the environment (sanitary state effect: GLMM: *χ*^2^ = 0.0023, *df* = 1, *P* = 0.96). This staying time however decreased over the successive foraging trips, most probably reflecting a reduced exploration of a better-known area and an improved foraging efficiency (trip’s rank effect: GLMM: *χ*^2^ = 65.2, *df* = 7, *P* < 0.001; see Supplementary Fig. 2).

Concerning the exploitation of the prey patches, the majority of the visits made to a foraging area (78%; n = 329 visits) resulted in the retrieval of a cold-killed fly. Interestingly, the decision of individual foragers to retrieve a prey was not influenced by the vicinity of sporulating items (sanitary state effect: GLMM: *χ*^2^ = 1.26; *df* = 1; *P* = 0.26). In total, a prey retrieval was as likely to occur when the ants visited the UFA (0.77; n = 173 visits) or the CFA (0.78; n = 156 visits). As expected, the sporulating items on the CFA were never collected by the foragers.

**Figure 2:**
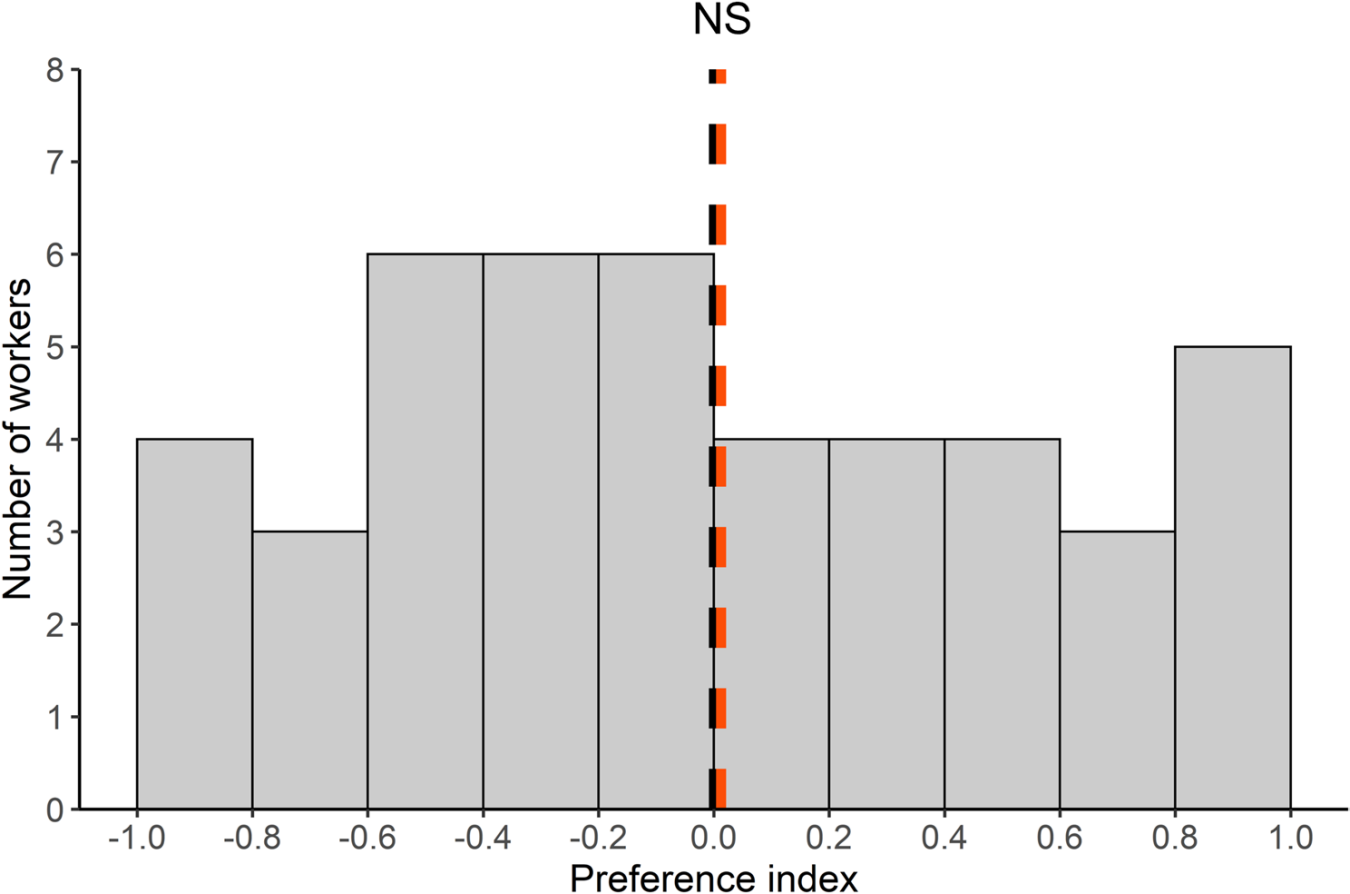
Individual experiment: distribution of the individual preference indices of foragers (n = 45). The dotted orange line represents the average of the individual preference indices and the dotted black line represented the value of 0 expected if contamination has no impact on foragers’ preference. Student's t-test; NS: non-significant.

Ant individuals may differ in their propensity to detect pathogen and to avoid a contaminated area. Therefore, we examined whether some individuals showed a preference to forage in one of the two areas. A preference index was calculated for each forager by averaging its scores when visiting the UFA (score of + 1) and the CFA (score of − 1). We found that the distribution of the individual preference indices followed a normal distribution (Shapiro-Wilk's test: SW = 0.96; *P* = 0.14, N = 45, Fig. 2). Some individuals (around 8%) exclusively foraged on the UFA while others visited only the CFA (around 11%). However, when considering all the tested individuals, the distribution of these preference indices was not significantly different from 0 (Student’s t-test: t = 0.14; *df* = 44; *P* = 0.89, N = 45). This means that, globally, the foragers were as likely to visit each of the two platforms regardless of the presence of sporulating items.

**Figure 3:**
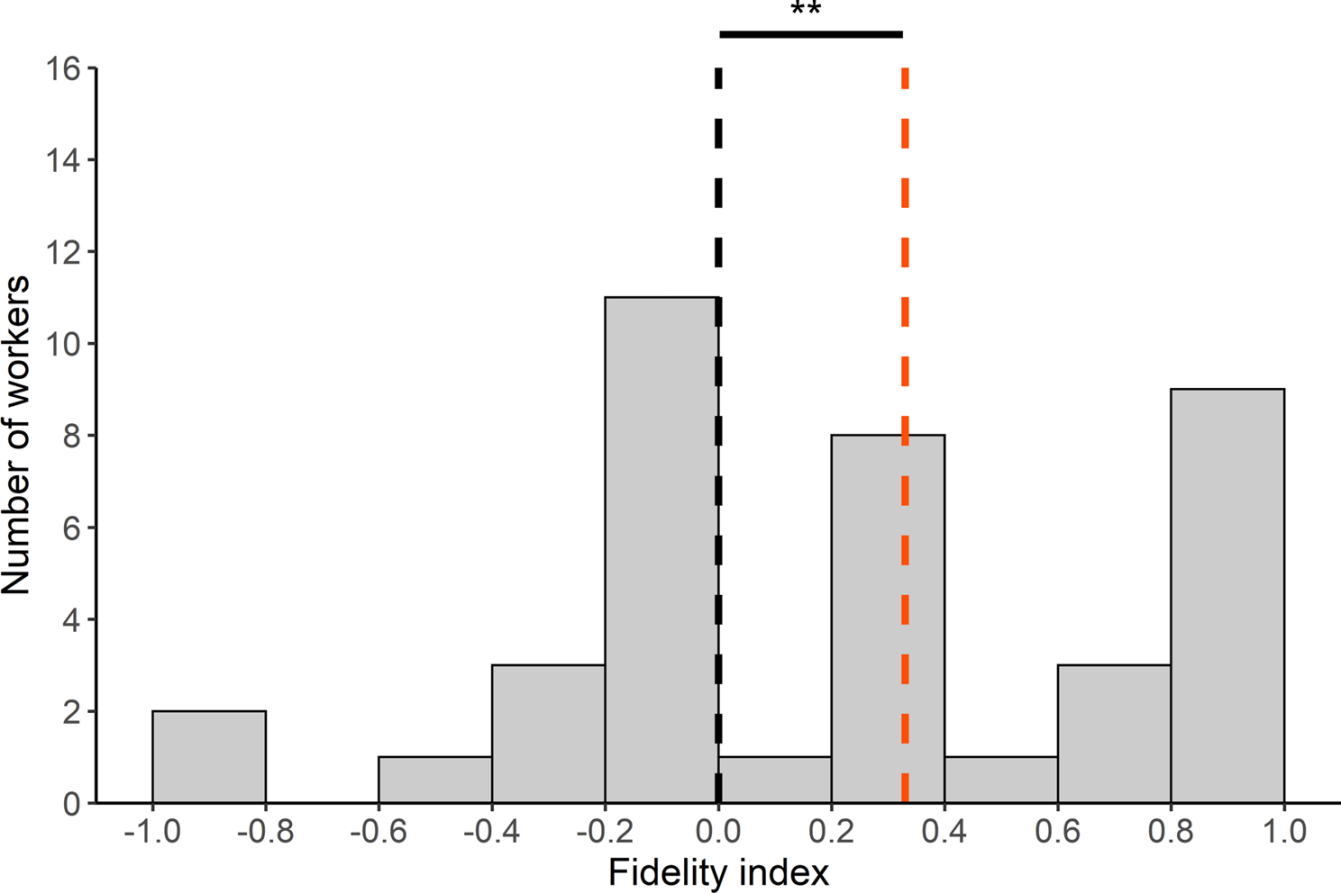
Individual experiment: distribution of the spatial fidelity indices for the foragers that performed several trips to the Y-maze (N = 39). The dotted orange line represents the median value of the observed individual fidelity indices and the dotted black line represents the value of 0 expected if the ants were as inclined to go toward the same platform as they were to switch between successive trips. One-sample Wilcoxon signed rank test; **: *P *< 0.01

**Figure 4:**
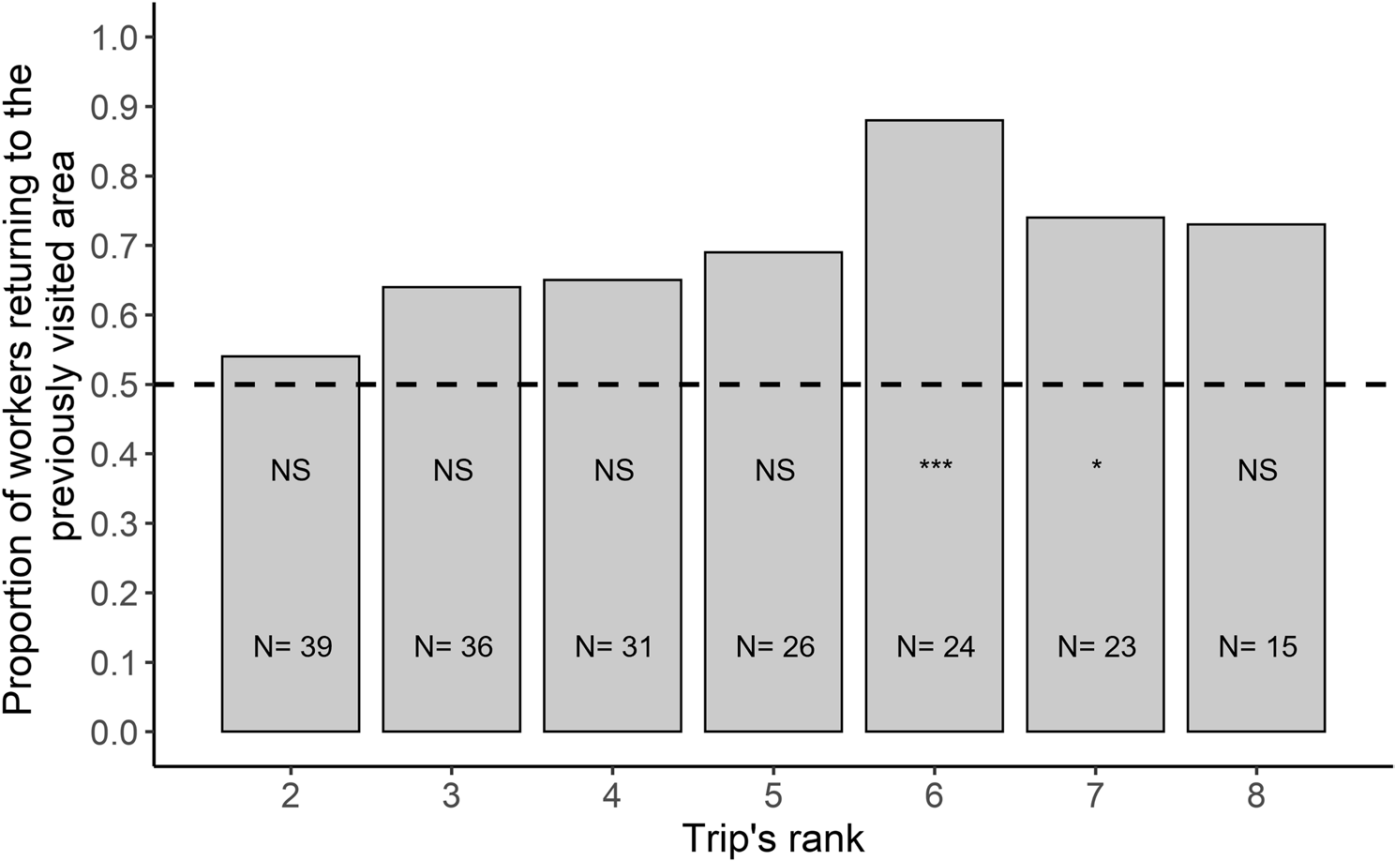
Individual experiment: proportion of foragers returning to the previously visited area as a function of the rank of the foraging trip. N values represent the number of ants for each trip's rank. A binomial test was carried out for each trip’s rank. Binomial tests; NS: non-significant, *:P ≤  0.05, ***:P ≤  0.001.

**Figure 5:**
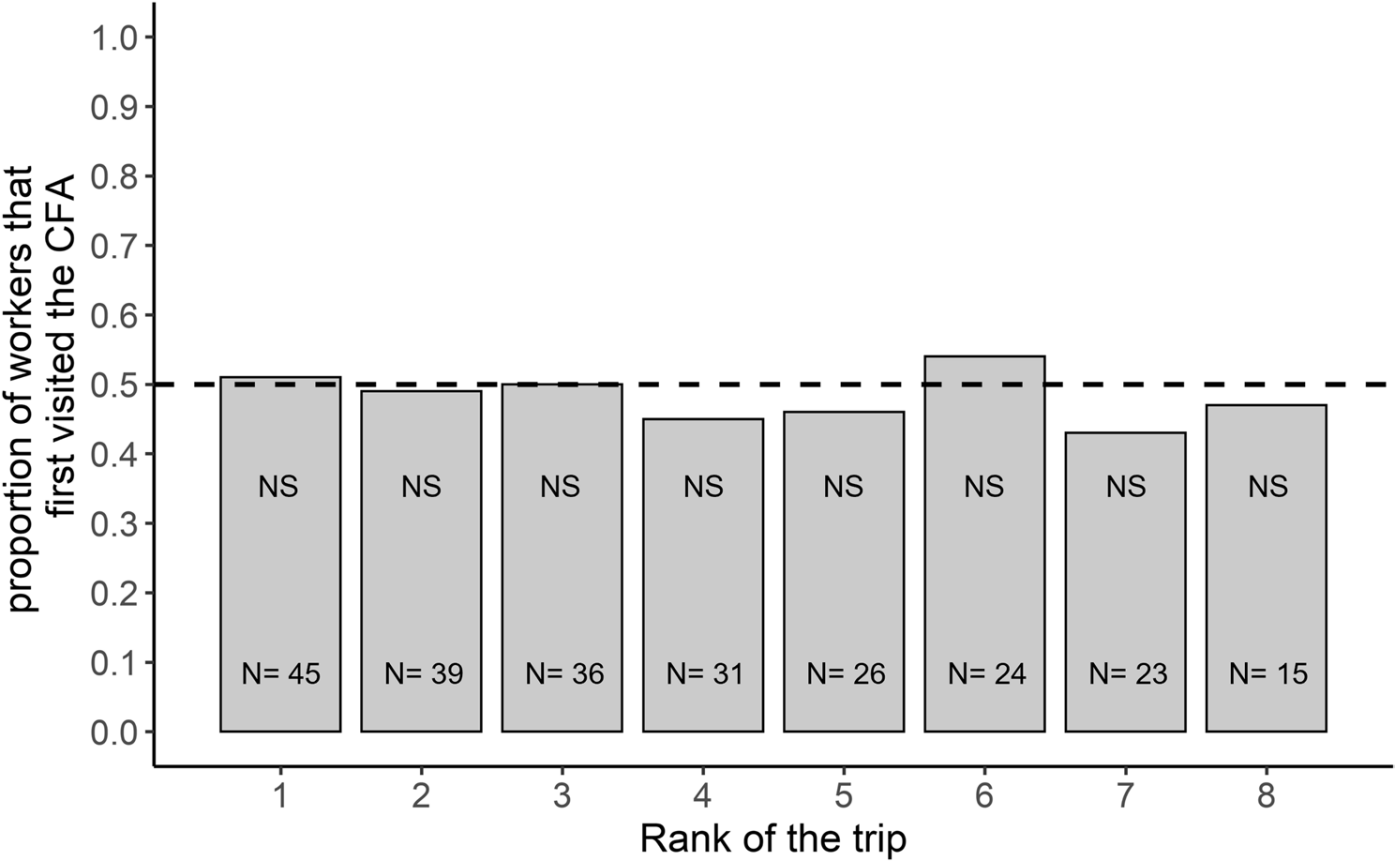
Individual experiment: proportion of foragers that first visited the contaminated foraging area (CFA) over the first eight trips. N values represent the numbers of ants for each trip's rank. A binomial test was carried out for each trip’s rank. Binomial tests; NS: non-significant.

We wondered whether a contamination of the environment had an impact on the spatial fidelity of individuals to a given area. We considered all the foragers that made multiple trips to the Y-maze. We examined the first two trips made by each forager to see whether they headed towards the same area as the one visited first or whether they started to explore the other area. Around half of the ants remained faithful to the same area while the other half switched from one platform to the other. Furthermore, foragers that returned to the same foraging area were as likely to be observed when they first visited the UFA (58%; N = 22) or the CFA (56%; N = 23). Thus, the propensity of foragers to return to the same foraging area was not influenced by its associated sanitary risk (sanitary state effect: GLMM: *χ*^2^ = 0.038; *df* = 1; *P* = 0.85). By taking into account all the trips (two trips or more) made by a forager, we estimated the spatial fidelity of each individual by counting the number of times it changed or sticked to the same area over two successive trips. We found that the fidelity indices of individual ants (N = 39 ants) were not normally distributed (Shapiro-Wilk's test: SW = 0.92; *P* = 0.012, N = 39) and that their median value was significantly higher than 0 (one-sample Wilcoxon signed rank test: one-tailed: V = 368, *P* = 0.0026, N = 39, Fig. 3). This suggests that some individual foragers showed a spatial fidelity to a foraging zone. This fidelity progressively emerged over time since the proportion of ants that kept on going to the same area, became significantly higher than 0.5 only on the 6th and 7th foraging trip (binomial test: *P* < 0.05, Fig. 4). Noticeably, exposure to sporulating items on CFA did not prevent ants to become faithful to a foraging area. Indeed, the fidelity index of an individual was not significantly correlated with the proportion of visits it made to the CFA (Spearman test: r_s_ = − 0.14; *P* = 0.4, N = 39; see Supplementary Fig. 3). Furthermore, the ants did not learn to avoid risky areas over time since the proportion of foragers first visiting the CFA never differed from 0.5 across their successive trips to the Y-maze (all binomial tests *P* > 0.05, Fig. 5).

### Collective foraging response

**Figure 6:**
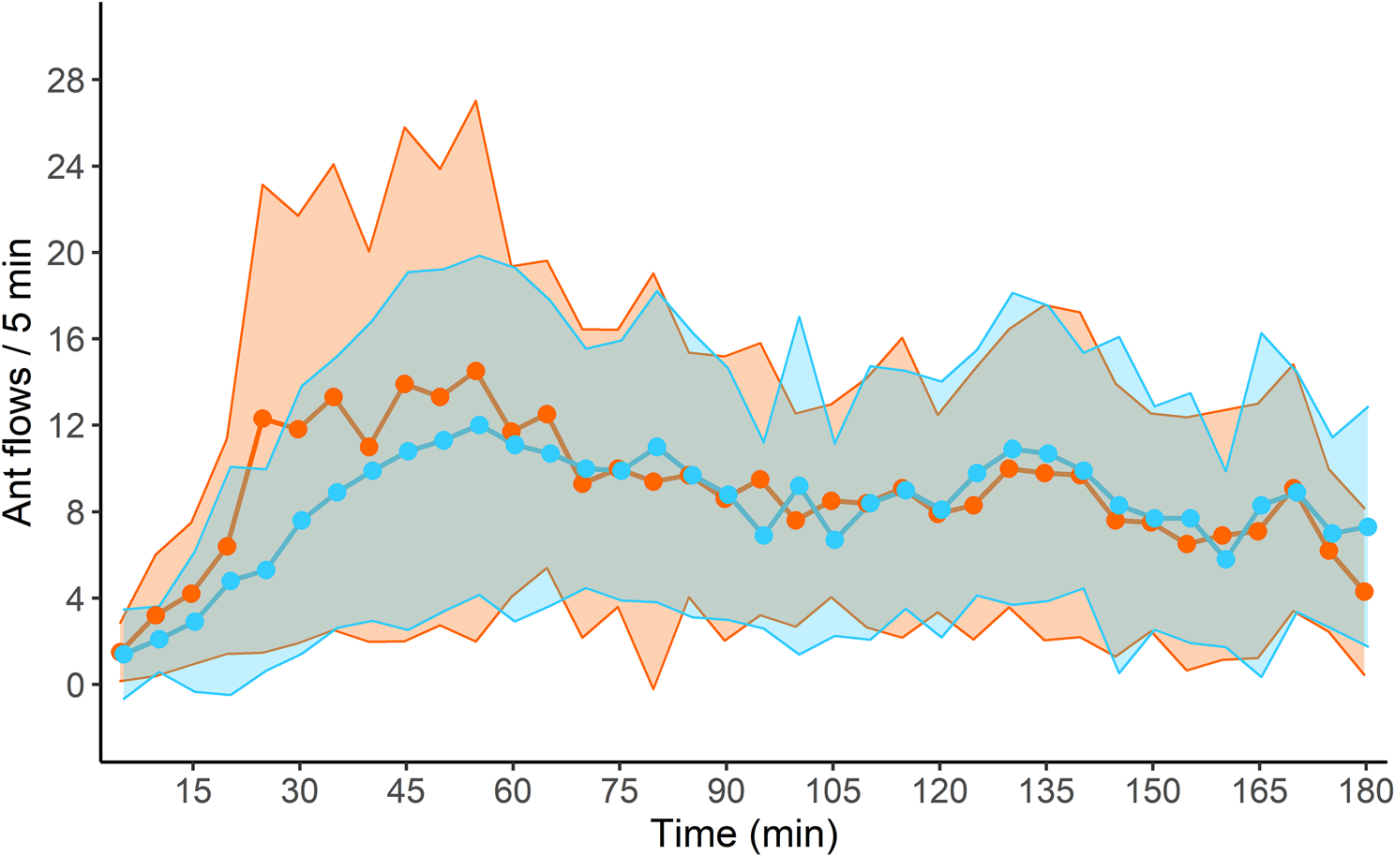
Collective experiment: ant flows arriving at the UFA (in blue) and the CFA (in orange) every 5 min during the 3-h experiment. Values were averaged over the number of workers entering the foraging area per 5 min of observation. Circles and shadings represent the mean ± standard deviation, respectively (N = 10 colonies).

We compared the 5-min flows of ants that reached each foraging area. The flows of ants towards each platform increased rapidly during 1 h and then decreased until the end of the experiment. The flows of ant foragers significantly changed over time (time effect: GLMM: Wald test: *χ*^2^ = 319; *df* = 35; *P* < 0.0001; Fig. 6), but was no influenced by the sanitary risks associated to the foraged platform (sanitary state effect :GLMM: *χ*^2^ = 2.80; *df* = 1; *P* = 0.094). Thus, colonies mobilized a similar number of workers towards the two patches of prey regardless of the presence of sporulating items in their vicinity.

**Figure 7:**
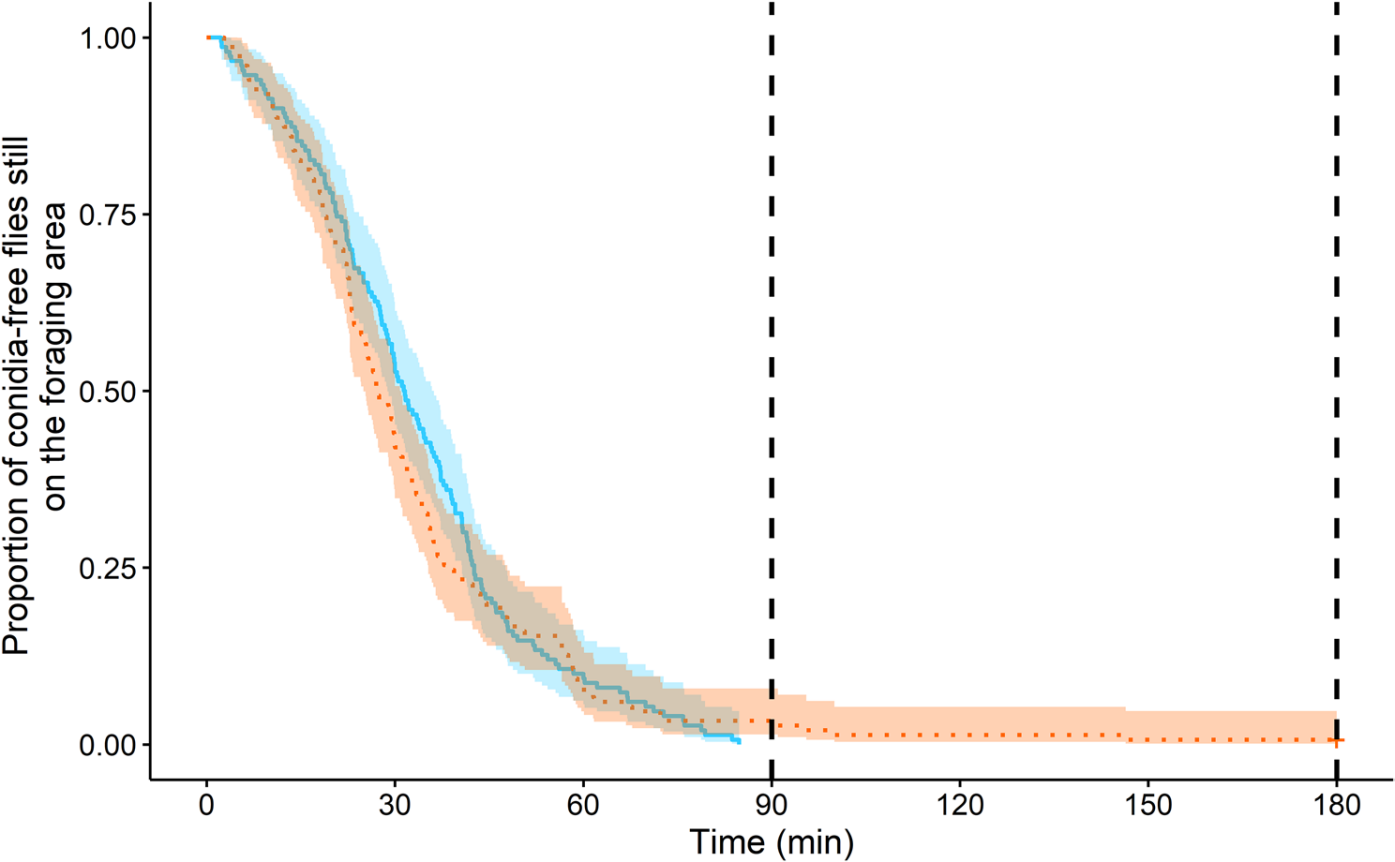
Collective experiment: the proportion of dead flies still on the UFA (in blue) and on the CFA (in orange) as a function of the time (min). The coloured shadings surrounding lines represent the 95% confidence interval. Curves were drawn by pooling all the conidia-free prey given on each platform for the ten tested colonies (N = 150 dead prey). The two vertical dashed lines indicate the half (i.e., 1 h 30) and the end of the experiment (i.e., 3 h).

Only conidia-free prey were retrieved from the UFA and CFA. The dynamics of prey retrieval did not significantly differ depending on the sanitary risks of the foraging environment (Log Rank test: *χ*^2^ = 0.4; *df* = 1; *P* = 0.5, Fig. 7). The first conidia-free prey were taken after a median time of 9 min on the UFA (m = 9 min [4; 14]; N = 10) and 6 min on the CFA (m = 6 min [5; 7]; N = 10). The last prey were taken after a median time of 47 min (m = 47 min [40; 65]; N = 10) and 51 min (m = 51 min [38; 66]; N = 10) for the UFA and CFA, respectively. Therefore, the prey retrieval time did not significantly differ according to the contamination of the foraging area (Wilcoxon signed rank test UFA VS CFA: first prey: W = 36, *P* = 0.43; last prey: W = 17, *P* = 0.32).

Fourteen days after the end of the experiments, the mortality of workers was negligible in all colonies. We found a median number of 12 [5; 17] dead workers per colony, representing around 6% of the workers’ population. None of these 118 corpses showed any sign of infection by *Metarhizium brunneum* fungus.

## Discussion

Once brought by foragers inside the nest, pathogen-bearing food items become a sanitary threat for the whole colony and may eventually lead to a disease outbreak. Therefore, foragers usually avoid to retrieve sporulating insects (in *Myrmica rubra*^48^), sporulating nestmates (in *Formica rufa*^51^) or items covered with fungal conidia (in *Polyrhachis dives*, *Messor barbarus*, *Acromyrmex echinatior*, *Formica rufa*^50^). The level of pathogen avoidance however differs according to the feeding ecology of the ant species^50^. Species that store food in their nest are more likely to avoid retrieving items covered with conidia than species that quickly consume the collected food. The stage of development of the pathogens can also influence the foraging behaviour of ant workers. While *Myrmica rubra* foragers may retrieve an insect that was artificially covered with a huge amount of conidia after its death, they consistently avoid to take a sporulating prey that bears conidia due to succumbing from infection^48^. Far less is known about the ability of workers to detect sanitary risks that are not directly associated to the food itself but instead, to the environment where foraging is taking place. We found that the vicinity of infectious sporulating items does not alter the willingness of *Myrmica rubra* ants to retrieve conidia-free prey. Likewise, we found no influence of sanitary risks on the collective choice of a foraging area nor on the staying time of ant workers on it. These results go against the expected avoidance and limitation of time spent by foragers on a potentially health-hazardous environment.

As regards to the spatial fidelity to a foraging zone, it increased gradually with the number of trips made by the forager. This finding confirms the short-term spatial memory that *M. rubra* foragers can develop toward food sources in the field^52^ or towards locations where conspecific corpses are deposited in the case of necrophoresis^53^. Quite unexpectedly, the presence of sporulating items put in the vicinity of palatable prey had no impact on the willingness of workers to return to a food source, with ants being as likely to develop a spatial fidelity to a contaminated foraging area as to a conidia-free area. Future studies are however needed to investigate whether some foraging environments, e.g. aphid colonies, that persist for weeks with some associated sanitary risks, may alter the spatial fidelity of ants or may even induce a progressive avoidance over longer time-scale.

Overall, our study adds to the growing body of evidence that ant responses to pathogens present in the environment can be ambiguous and highly variable, ranging from an avoidance^50^ to certain forms of attraction^54,55^. For instance, some *M. rubra* colonies prefer to dig their nest in a conidia-free substrate while others prefer to excavate it in a soil contaminated by *M. brunneum* conidia^56^. In nature, opportunistic entomopathogens such as *Metarhizium* or *Beauveria* fungi are ubiquitously found below and over the soil surface^57^. Terricolous ants like *M. rubra* are thus frequently exposed to conidia of *Metarhizium* fungus, both when staying inside their nest^58^ and when foraging in the outside^59^. In this context, a systematic avoidance of fungal parasites would be unsustainable for *M. rubra* colonies because it would drastically reduce the number of biotopes as well as the size of the ecological niche still available for nesting or foraging.

To counter the risks of getting infected when exploiting food in an environment contaminated by pathogens, ant foragers are expected to display hygienic behaviours. However, in several ant species, grooming events have been reported only when ant’s body was contaminated by very high doses of entomopathogenic fungi (above 10^5^ conidia^60,61^ but see^62^). In the present study, the incidental contamination of foragers’ body by about 30,000 conidia per individual did not trigger more self-grooming responses and led to low rates of mortality with no dead nestmates showing any signs of fungal infection. This suggests that self-or allo-grooming of foragers may have taken place inside the nest, thereby contributing to reduce the amount of conidia on their body to sublethal doses.

The lack of fungus-induced mortality as well as the lack of avoidance of a contaminated environment on behalf of *M.rubra* foragers support the “field realism criticism” raised by Loreto and Hughes^57^. Most estimates of fungus pathogenicity are based on ant’s mortality under laboratory conditions, in which extremely high doses of fungal conidia were often delivered to the ants by unrealistic modes of contamination such as a direct application on the insect body. More biological relevance and closer-to-nature conditions are expected from protocols of exposure to pathogens. When foragers were free to avoid sporulating items (as in this study) or when conidia were applied on soil instead of a more artificial substrate^63^, ant colonies suffered from far lower rates of infection and mortality. Interestingly, a 1-year field study^64^ on various species of leaf cutting ants also found less than 0.1% of live individuals being infected, despite the high levels of *Metarhizium* fungus in the soil inhabited by the colonies. Finally, there is no record of natural epizootic events in ant colonies and natural infections are rather isolated and very rare (reviewed in ^57^). Taken all together, these studies suggest that the complexity of a more realistic environment makes the actual exposure of ants to pathogens and the incurred sanitary risks much lower than commonly reported in laboratory studies. Furthermore, the social life of ants also contributes to their resistance against microparasites. Pathogenicity is typically a sigmoidal function with threshold doses leading towards a maximal mortality^65^. Cooperative behaviour between workers inside the nest may contribute to keep the amount of parasites below the threshold of a significant pathogen-induced mortality. This can be achieved by a careful inspection and grooming of workers that returned to the nest^66,67^. Besides the active removal of parasites by mutual grooming, frequent contacts of foragers with nestmates could lead to a passive transmission of conidia and hence to dilution effects reducing the per-ant capita exposure to sublethal doses of pathogens^65^. One may even consider the possibility of delayed benefits such as certain forms of acquired immunity. This would confer an increased specific individual resistance and a higher survival rate after a first exposure to pathogens (bumble bees^68^; termites^69^; ants^70,71^). Nevertheless, immune priming in ants is still under debate^72,73^ and awaiting to be investigated in *Myrmica rubra* species.

It is commonly accepted that parasites and pathogens represent a potential threat shaping the life history traits of the host species. Whether generalist entomopathogenic fungi such as *Metarhizium* actually act as a strong selective force on ant societies is another matter, for which the answer needs to be mitigated. As regards to foraging, several ant species have evolved efficient and non-specific behavioural strategies that limit nest contamination such as forgoing the retrieval of sporulating dead insects^48,51^ or fastly discarding wastes^42^. On the other hand, we found that the contamination of the environment where foraging takes place does not influence the behaviour of ants. The vicinity of the fungal pathogens near exploited food sources does not deter ant workers nor alter their spatial fidelity to these foraged areas. This unveils that more naturalistic conditions for ants to get infected, such as walking close to conidia-releasing items, expose the ants only to sublethal doses of pathogens. The resulting sanitary risks can be too low for ants to display a systematic avoidance of contaminated zones, what would be costly in terms of restriction of areas still available for foraging. The survival, the susceptibility and the behavioural response to generalist pathogens is known to differ among ant species, namely according to their feeding ecology^50,60^. Here, we suggest that the circumstances under which ants encounter microparasites also contributes to the high variability of their avoidance strategy. In the case of foraging, the presence of generalist fungal conidia near food sources seemed to be more of a daily nuisance to the ants, rather than a major threat for the survival of ant societies.

## Material and methods

### Biological material

*Myrmica rubra* is a polygynous and monomorphic ant species living in temperate zones of Europe. This species can be found in a wide range of environments, with a preference for semi-humid habitats such as stumps of dead trees, loose soil under stones or meadows^74,75^.

*Metarhizium brunneum* is a generalist entomopathogenic fungus commonly present in the soil, including in the nesting sites of *M. rubra* ants^59^. Once in contact with the insect host, *M. brunneum* conidia attach themselves to its body surface and develop hyphae that pierce its cuticle by releasing fungal enzymes. The fungus mycelium then grows inside the insect body, what ultimately causes its death. Several days after the host death, the fungus begins to sporulate over the corpse and produces new mature conidia that will be released in the environment^76^.

Colonies of *M. rubra* were collected in June 2019 in Falisolle (50° 25′ 11.99″ N; 4° 37′ 50.41″ E) in Belgium. They were placed in plastic boxes (40 × 25 × 7 cm) whose edges were coated with Fluon to prevent ants from escaping. We kept colonies in the laboratory at a constant temperature of 21 ± 2 °C, at 50 ± 5% relative humidity and at a photoperiod of 12L–12D. The nests were made of glass test tubes with a water reservoir and covered with a red plastic filter. We supplied colonies ad libitum with water and sugar (0.3 M sucrose solution) and with mealworms (*Tenebrio molitor*) twice a week as a source of proteins.

As a food source, we used *Drosophila melanogaster* fruit flies (“vestigial wings” phenotype). Fruit flies were reared on a home-made food substrate (79% applesauce, 3% oat bran, 12% mashed potatoes in snowflakes, 5% white vinegar) at a room temperature of 21 °C and a 50% relative humidity. We dropped raffia strings on the mixture in order to facilitate the pupation of larvae. We renewed the food substrate once per month.

As a source of contamination, we used sporulating fruit flies. They were obtained by vortexing living flies in an Eppendorf with a sporulating fly cadaver previously (four times during 5 s at 1500 RPM according to protocol in Ref.^77^). The conidia-covered flies were then isolated in a plastic box with water and sugar, with death occurring approximately 4–5 days later. Their corpses were collected on the day of the insect death, washed with ethanol and then rinsed with distilled water according to the Lacey method^78^ to prevent the growth of other opportunistic microorganisms. We then placed the fly corpses on a moist filter paper in Petri dishes hermetically sealed and kept them in a thermostatic cabinet at 25 °C for a period of 2–7 days until the fungus started to sporulate.

### Experimental setup

The experimental set-up consisted in a Y-maze leading to two separate platforms. The Y-maze was composed of a common trunk (10 × 1.5 cm) and two branches (8 × 1.5 cm), forming an angle of 60° and each leading to a foraging area (9 cm^2^) where the prey patches were deposited. The ant nest was connected to the Y-maze by an access ramp (15 × 1.5 cm). A removable part (1.5 cm × 1.5 cm) was cut on the common trunk at 2 cm from the access ramp. When required for following up ant individuals, this removable part allowed to give access to a single worker at a time (see Supplementary Fig. 4). During the entire experiment, the Y-maze was recorded from above using a webcam Logitech Pro HD C920.

In order to facilitate ants’ navigation and memorization of food location, we added visual cues to the experimental setup: (1) a white LED light source was centred between the two branches of the Y-maze and (2) two black pictograms of identical area (2.25 cm^2^) representing a triangle or a square, were placed on the side of each branch.

On the platform referred as the uncontaminated foraging area (UFA), the food source consisted in 15 cold-killed and conidia-free flies that were evenly distributed over the area. On the other platform referred as the contaminated foraging area (CFA), the food source also consisted in 15 cold-killed and conidia-free flies but they were deposited close to 10 *M. brunneum* sporulating flies. These ten sporulating flies were arranged alternately and separated from each other by about 2 mm. This separation distance prevented the body of conidia-free flies from being contaminated by sporulating corpses. As the number of palatable items was the most likely to influence ants’ foraging behaviour, we prioritize equality in the number of conidia-free prey introduced on each area. Besides, the lack of retrieval of sporulating flies in a previous study^48^ indicates that they are no longer perceived as prey by the ants, thereby minimizing any foraging bias due to the presence of ten additional sporulating flies on the CFA.

The ten sporulating items on the CFA represented approximatively a level of contamination of 10^7^ conidia. Indeed, the number of conidia covering the body of a sporulating fly was estimated to around 10^6^ spores following the procedure described in Ref.^49^. In order to prevent any unwanted contamination of the UFA by conidia dispersed through air flows, all the experiments were carried out inside a closed box under still air conditions. Besides, the renewal of papers over the Y-maze between successive trips as well as the very few number of ants that explored first the CFA and then the UFA (6% out of 287 trips) made a contamination of UFA due to spores incidentally transported by walking foragers very unlikely.

### Individual response to fungus-contamination of foraging areas

Experiments were carried out on ten colonies composed of 200 workers (80 foragers and 120 internal workers), one queen and 50 larvae (second and third stages). In order to motivate foragers to search for prey, colonies were starved for 3 days before the experiment by depriving them from mealworms and sugar sources.

The day before the experiment, 25 individual foragers that were wandering outside the colony were gently removed. After being chilled at − 10 °C for 30 s, they were marked with a unique colour code on the abdomen (two dots of enamel paint marker Edding™ 8750). Once marked, the ants were isolated for 3 h to avoid paint removal by allo-grooming nestmates, and then were reintroduced inside their colony.

For the individual experiments, a single marked forager was allowed to enter the Y-maze at a time and was followed over its consecutive trips to the Y-maze. The experiment started as soon as a marked ant entered the Y-maze and reached the bifurcation leading to the two foraging platforms. The experimenter then raised the removable bridge to prevent any further passage of other workers. The experimenter identified the ant in real time thanks to its colour code. Once the ant had visited a foraging platform and had returned to the common trunk of the bridge, the experimenter allowed this ant to exit the Y-maze by placing again the removable section of bridge (see Supplementary Fig. 4).

At most 5 min were needed for the ant to reach one of the two platforms. Then, it was allowed, for at most 20 min, to inspect the prey source and to exit the Y-maze. Preliminary experiments showed that this amount of time was sufficient for 93% of the individuals (N = 13) to freely explore the foraging platform. We allowed each marked ant to return on the Y-maze as many times as it wanted. The consecutive trips made by the same ant were followed for a maximal duration of 90 min after its first entry on the maze.

During the experiment, the observer noted which platforms were explored by the marked ant over its successive foraging trips on the Y-maze. We defined a trip as the exploration made by the ant from its entry on the Y-maze until its exit. We defined a visit as the exploration made by the ant over one of the two platforms. A worker could thus do several visits during the same trip when it explored several times either one or the two platforms before exiting the Y-maze. The number of visits to the conidia-contaminated area allowed to assess whether sanitary risks alter the exploration as well as the spatial fidelity of workers to a foraging site. Likewise, the number of trips to the Y-maze quantified the persistence of ant individuals in being engaged in a foraging activity. We also examined whether the presence of sporulating items on the CFA reduces the level of food exploitation -here the number of retrieved flies. A fly was considered as being retrieved when being transported until the end of the Y-maze.

In order to exclude any effect of trail pheromone on ants’ orientation and choice of a foraging platform, the common section as well as the branches of the Y-maze were covered with a white paper which was renewed between successive observations of individual foragers. Furthermore, in order to avoid any left–right bias on ants’ choice of a food source, the location of the contaminated foraging platform was switched for half of the ten replicates.

Each experiment lasted at most 3 h. Every time a worker retrieved a prey, we re-adjusted the number of palatable flies on the platform in order to prevent any bias due to food depletion.

In total, we followed up 45 marked ants over the ten tested colonies (see Supplementary Table S1).

### Collective response to fungus-contamination of foraging areas

Experiments were carried out on ten colonies starved for 3 days and having access to the same Y-maze setup as the one described above. However, in this case, all the foragers had free access to the Y-maze for the whole duration of the experiment. As previously described, 15 cold-killed conidia-free prey were placed either on a pathogen-free platform (UFA) or close to ten sporulating items (CFA). Once the colony was connected to the Y-maze, workers were free to explore it, to forage on the platforms and to collect dead flies for a total duration of 3 h. The number of available conidia-free prey was not re-adjusted during the experiment.

We recorded the Y-maze throughout the experiment with a webcam (Logitech Pro HD C920) to observe ants’ behaviour. On video recordings, we quantified the dynamics of prey retrieval as well as the flows of ants, i.e. the number of foragers entering each foraging area over successive 5-min time intervals.

After the end of the experiment, we measured the daily mortality of workers in each colony during 14 days. We washed the ant corpses (according to Lacey's method^78^) and placed them in a temperature-controlled cabinet in order to check whether *M. brunneum* fungus had caused the ants’ death.

We roughly assessed whether the ants having explored the CFA had their body contaminated by conidia. To this aim, we let ten workers exploring CFA during 2 min and 13 s (median staying time during the first trip on CFA). Then, we put these workers in an Eppendorf containing 1 mL of Triton X 0.05% solution and vortexed it five times for 10 s. We removed the workers and centrifugated the solution containing the conidia during 10 min at 1600 rpm. After removing the supernatant, we added 500 µL of Tween 20 solution and we scattered the pellet by vortexing it. Finally, by counting the number of conidia with a Thomas’s cell under a microscope, we estimated that the foragers’ body was covered by around 30,000 conidia per capita.

### Statistical analyses

All data were analysed with R software (version 4.0.2)^79^ and all tests were two-tailed (excepted when specified) with a significance level of alpha = 0.05. Data were expressed as medians, 1st and 3rd quartiles (median [Q1, Q3]), except for the flows of ants towards a foraging area which were averaged (mean + SD). All the figures were achieved with the package “*ggplot2*”^80^.

#### Individual response of foragers

To check whether external factors in the laboratory had biased the orientation of ants, we tested whether the proportion of workers that first discovered either the left or the right platform differed from random by using a binomial test. Likewise, we examined whether ants were attracted or repelled from a distance by sporulating items on CFA by analysing with a binomial test whether the proportion of foragers that first explored the UFA or the CFA differed from 0.5. We also compared with a Mann–Whitney test the total duration of grooming events performed by foragers while first visiting the UFA or the CFA.

We checked whether the level of exposure to sporulating items altered the willingness of workers to stay engaged in foraging. We thus used a Spearman test that correlated the proportion of visits made by each worker toward the CFA to its total number of trips on the Y-maze. We also investigated whether the time spent by foragers on a foraging platform depended on its contamination and changed across the successive trips to the Y-maze. To this aim, we carried out a general linear mixed model (GLMM) analysis with the sanitary state of the platform (UFA vs CFA) and the rank of the trip (1st to 8th trip done by the ant) as fixed factors and the individual ID nested in colonial ID as random factors (quasi-Poisson distribution) (see Supplementary Table S2). The subsequent trips were not taken into account for these statistical tests because a too small sample size of foragers (N < 10) did nine trips or more. As regards the prey retrieval by individual foragers, we also used a GLMM to assess whether the sanitary risks influenced the number of visits that ended with the retrieval of a prey. The sanitary state of the environment (UFA vs CFA) was considered as a fixed factor and the individual ID nested in colonial ID as random factors (quasi-binomial distribution). For these two GLMM analyses, we used ANOVAs with repeated measure (with glmmPQL R package “*MASS*”^81^). To study whether individuals headed toward the same foraging area during their second trip, we performed a GLMM (binomial distribution) by including the sanitary state (UFA vs CFA) as a fixed factor and the individual ID nested in colonial ID as random factors. This GLMM was carried out with the R-packages “*lme4*”^82^ and “*lmerTest*”^83^. We checked that data met the models’ assumption and showed no under or over-dispersion based on model deviance/degrees of freedom values. When significant, we performed pairwise comparisons with Tukey adjustment of p values, using the R-packages “*emmeans*”^84^ and “*multcomp*”^85^.

For each tested individual (N = 45), we calculated a preference index that reflected its propensity to forage on one of the two platforms. This preference index was calculated as follows: a score of 1 was allotted to each visit to the UFA, a score of − 1 to each visit to the CFA and these scores were averaged on all the visits made by the forager. A preference index close to − 1 or to 1 means that the individual visited mainly the CFA or the UFA respectively. A preference index of 0 means that a worker equally visited both foraging areas. As the distribution of preference indices met the normality conditions (Shapiro-Wilk’s test), we performed a Student's t-test to compare it with a normal distribution centred on a theoretical value of 0 (corresponding to a no-preference).

We also investigated whether individual foragers developed a spatial fidelity to one foraging area regardless of its sanitary state. For each new trip made by a forager on the Y-maze, we observed whether the first-visited platform was the same as the one it had left on the previous trip. If the ant successively visited the same platform, we assigned a score of 1 and if the ant switched to the other one, we assigned a score of − 1. We calculated the fidelity index of an individual by averaging the scores over all the visits made by the individual. A fidelity index of − 1 means that the ant systematically shifts from one foraging area to the other while an index of 1 accounts for an ant being perfectly faithful to a single platform. As the fidelity indices were not normally distributed, we used an one-sample Wilcoxon signed rank test (one-tailed) to check whether the fidelity indices of individual foragers were higher than the theoretical value of 0 expected if the ant was as likely to return to the same platform as to switch to the other one. Since exposure to the CFA may alter the spatial fidelity of foragers, a Spearman correlation test correlated the fidelity indices of workers and the number of visits they made on the CFA. We also used two binomial tests to check whether (1) the proportion of workers returning to the previously visiting foraging area as well as (2) the proportion of workers that first visited the CFA differed from 0.5 on each of the first eight trips to the Y-maze.

#### Collective foraging response

We measured the flow of ants arriving at either the CFA or the UFA every 5 min for 3 h of experiment. These flows were analysed with a GLMM (negative binomial distribution) by including the sanitary state of the area as well as the time as fixed factors and the colony ID as a random factor (see Supplementary Table S2).

Changes over time in the number of flies still available on each platform were compared for UFA and CFA conditions by using a log-rank test (with the R package “*survival*”^86^). We also compared the retrieval time of the first prey as well as the last prey between the UFA and CFA by using a Wilcoxon signed rank test.

## Supplementary Information

Supplementary Information.

## Data Availability

Data used in this article are available in the Zenodo Digital Repository: https://doi.org/10.5281/zenodo.5510628.
