# Supplementary material for: A fungus infected environment does not alter the behaviour of foraging ants
Source: Sci Rep. 2021 Dec 7;11:23573. doi: 10.1038/s41598-021-02817-8 (PMC8651729; doi:10.1038/s41598-021-02817-8)
Supplement: Supplementary file 1 — Supplementary Information. [file 41598_2021_2817_MOESM1_ESM.docx]

Supplementary materials for :

**A fungus infected environment does not alter the behaviour of foraging ants**

H. Pereira, R. Willeput, C. Detrain

corresponding author : Hugo Pereira ([hugopereira_54@hotmail.fr](mailto:hugopereira_54@hotmail.fr))


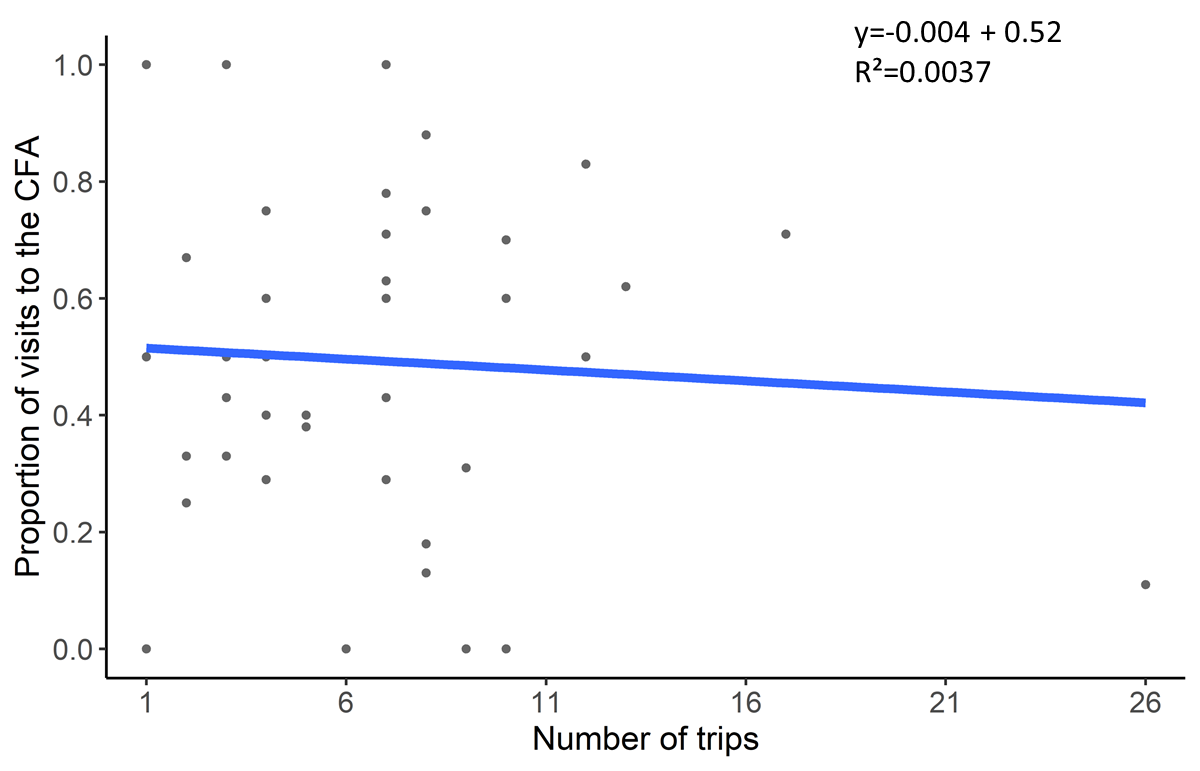


**Figure S.1:** Proportion of visits to the contaminated foraging area (CFA) as a function of the total number of trips made by an individual forager (N=45).


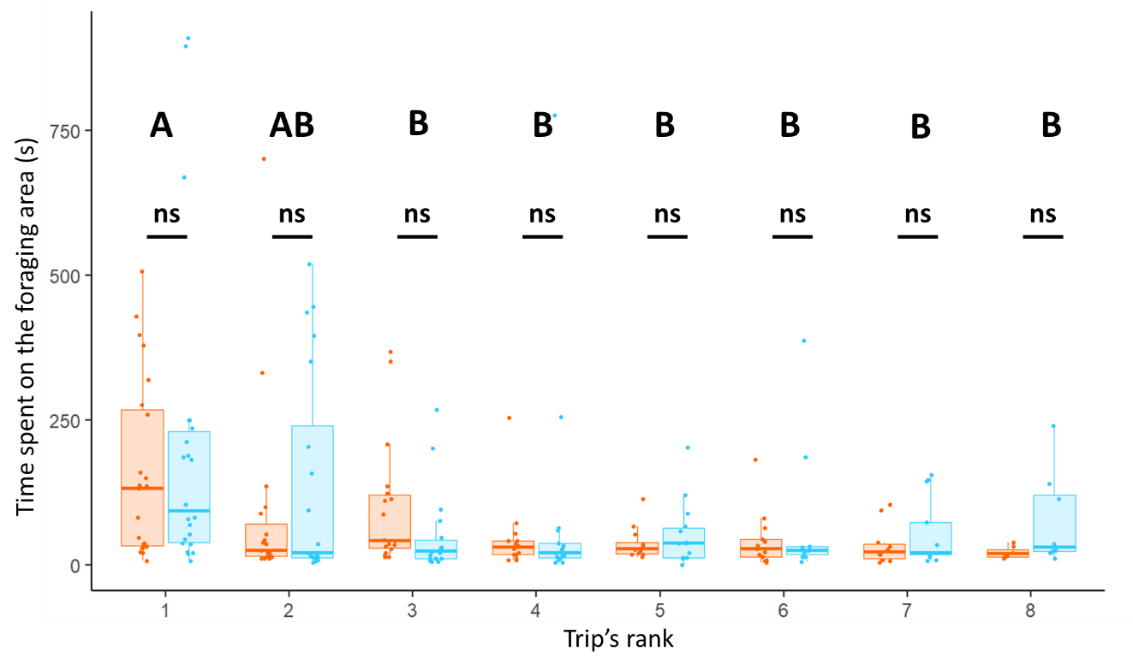


**Figure S.2:** Time spent by ants on the uncontaminated (blue) or contaminated (orange) foraging area depending on the rank of the trip. The horizontal bar within the boxes represents the median; the upper and lower boundaries of the boxes represent, respectively, the 75th and 25th percentiles, while the whiskers extend to the smallest and largest values within 1.5 box lengths. GLMM showed that the time spent by ants on the foraging areas did not depend on their sanitary states (GLMM: *ꭓ*²=0.02, *df*=1, *p*=0.96). For each condition, p-values are given for pairwise comparisons between observation sessions (NS = non-significant, *P* > 0.05). However, the time spent by ants on the foraging areas depended on the trip’s rank (GLMM: ꭓ²=65.2, *df*=7, *P* < 0.001). Trips sharing a common letter were not significantly different.


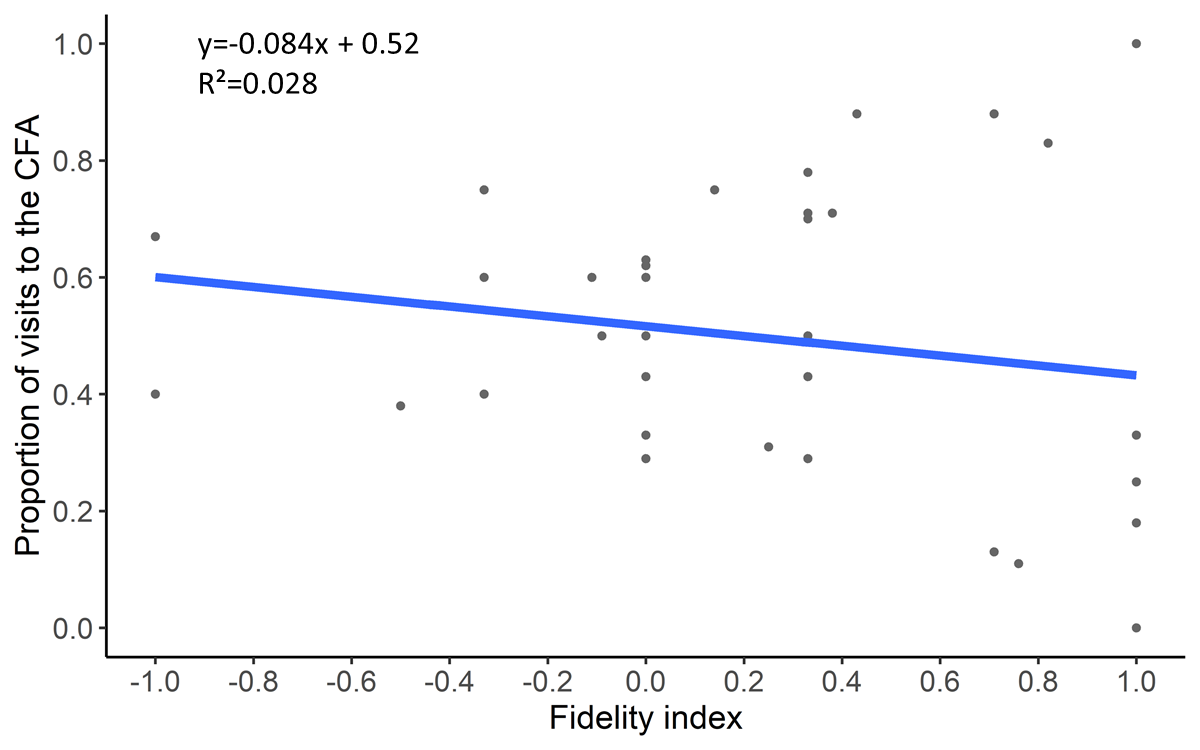


**Figure S.3:** Proportion of visits to the contaminated foraging area (CFA) as a function of the fidelity index of the individual (N=39).


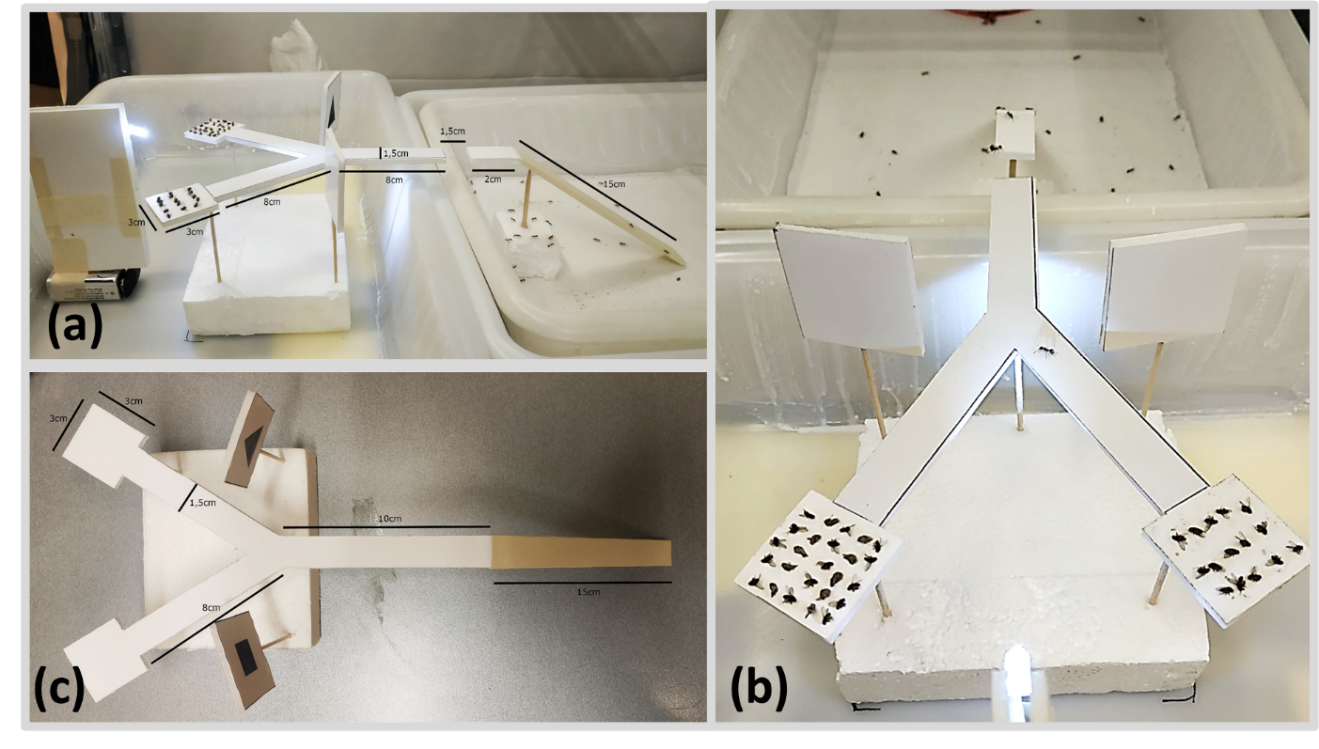


**Figure S.4** : Picture of the Y-maze used for the individual responses taken from (a) the side and from (b) above. Picture of the Y-maze used for (c) the collective experiment. Photos by Romain Willeput. Figure was created by using Microsoft 365 PowerPoint version 2108 (https://www.microsoft.com/en-us/microsoft-365/powerpoint).

**Supplementary Table S1**: Number of foragers tested individually in each colony.

| Colonies | Number of marked foragers used in the individual experiment |
| --- | --- |
| A | 2 |
| B | 4 |
| C | 4 |
| D | 3 |
| E | 7 |
| F | 3 |
| G | 7 |
| H | 5 |
| I | 5 |
| J | 5 |

**Supplementary Table S2**: Description of statistical models used in the manuscript. The random factors are written in *italics*.

| Experiment | Variable analysed | Factors | Family |
| --- | --- | --- | --- |
| Individual foraging experiment | **Time spent on the foraging platform** | Sanitary state of the foraging area  + Rank of the trip (first to eighth) + *Colonial ID/Individual ID* | GLMM: quasipoisson distribution |
|  | **Proportion of trips with a prey retrieval** | Sanitary state of the foraging area  + *Colonial ID/Individual ID* | GLMM: quasibinomial distribution |
|  | **Proportion of foragers returning to the same foraging area during their second trip** | Sanitary state of the foraging area  + *Colonial ID/Individual ID* | GLMM: binomial distribution(“probit function”) |
| Collective foraging experiment | **Number of workers entering the foraging platform** | Sanitary state of the foraging area  + time + *colonial ID* | GLMM: negative binomial distribution |
